# Annual Meeting of the American Medical Association

**Published:** 1851-06

**Authors:** 


*Annual Meeting of the American Medical Association.*

Circumstances prevented us, much to our regret, from attending the Annual Meeting of the American Medical Association, recently held at Charleston, S. C.; and the accounts which, up to this time, we have received, embrace only a report of the last day's proceedings, and brief notices of the session, in the New-York Medical Gazette, and the Boston Medical and Surgical Journal.

Somewhat over two hundred delegates and members were present at the meeting. Harmony and good feeling as usual prevailed, and the hospitality and attentions of our Charleston brethren were unbounded.

The officers elected for the present year are as follows:

Dr. Moultrie, of Charleston, S. C., President; Drs. George Hayward, of Boston, Mass.; Welford, of Petersburg, Va.; Arnold, of Savannah, Geo.; and J. B. Flint, of Louisville, Ky., Vice-presidents. Drs. De Saussure, of Charleston, S. C.; and Gooch, of Richmond, Va., Secretaries.

An important change in the constitution of the association was made, viz. articles providing for Standing Committees on Medical Science, Practical Medicine, Surgery, Midwifery, Medical Education, &c., were abolished. And, in the places thereof, a large number of committees are to report on special subjects designated by the Association.

The following are the resolutions submitted by Prof. George B. Wood, of Philadelphia, and adopted by the Association, which specify the subjects on which the committees are to report at the next annual meeting. In quoting the resolutions, we will append to each the chairman subsequently appointed, who, as will be seen by the last resolution, is to select two associates to constitute, with himself, the committee:

1st. Causes of the tubercular diathesis. Dr. D. F. Condie, of Philadelphia, chairman.

2d. Blending and conversion of the types of fever. Dr. S. H. Dickson, of Charleston, S. C.

3. The mutual relations of yellow fever and bilious remittent fever. Dr. James Jones of New-Orleans.

4th. Epidemic erysipelas. Dr. John B. Johnson, of St. Louis.

5th. Acute and chronic diseases of the neck of the uterus. Dr. Charles D. Meigs, of Philadelphia.

6th. Dengue. Dr. J. P. Jervey, of Charleston, S. C.

7th. The milk-sickness, so called. Dr. Daniel Drake, of Louisville, Ky.

8th. Endemic prevalence of tetanus. Dr. A. Lopez, of Mobile, Ala.

9th. Diseases of parasitic origin. Dr. George B. Wood, of Philadelphia.

10th. Physiological peculiarities, and diseases of negroes. Dr. R. D. Arnold, of Savannah, Geo.

11th. The action of water on lead-pipes, and the diseases which proceed from it. Dr. Horatio Adams, of Waltham, Mass.

12th. The alkaloids which may be substituted for quinia. Dr. Joseph Carson, of Philadelphia.

13th. Permanent cure of reducible hernia. Dr. George Hayward, of Boston, Mass.

14th. Results of surgical operations for the relief of malignant diseases. Dr. S. D. Gross, of Louisville, Ky.

15th. Statistics of operations for removal of stone in the bladder. Dr. James R. Wood, of New-York.

16th. Cold water dressings. Dr. Charles A. Pope, of St. Louis, Mo.

17th. The sanitary principles applicable to the construction of dwellings. Dr. Alex. H. Stevens, of New-York.

18. The toxicological and medicinal properties of our cryptogamic plants. Dr. Porcher, of Charleston, S. C.

19th. Agency of the refrigeration produced through upward radiation of heat as an exciting cause of disease. Dr. G. Emerson, of Philadelphia.

20th. Epidemic diseases of New-England and New-York. Dr. Worthington Hooker, of Norwich, Conn.

21st. Epidemic diseases of Pennsylvania, New-Jersey, Delaware and Maryland. Dr. John L. Atlee, of Lancaster, Penn.

22nd. Epidemic diseases of Virginia and North-Carolina. Dr. Robert W. Haxall, of Richmond, Va.

23d. Epidemic diseases of South Carolina, Georgia, Florida, and Alabama. Dr. Wm. M. Boling, of Montgomery, Ala.

24th. Epidemic diseases of Mississippi, Louisiana, Texas and Arkansas. Dr. E. H. Benton, New-Orleans, La.

25th. Epidemic diseases of Tennessee and Kentucky. Dr. Sutton, of Ky.

26th. Epidemic diseases of Missouri, Illinois, Iowa, and Wisconsin. Dr. Thomas Reyburn, of St. Louis, Mo.

27th. Epidemic diseases of Indiana, Ohio and Michigan. Dr. George Mendenhall, of Cincinnati, Ohio.

II. *Resolved*, That a Committee of Nomination be appointed, whose duty it shall be to nominate one Chairman for each of the above Committees.

III. *Resolved*, That each of the Chairmen thus nominated shall select, at his earliest convenience, the members of the Association to complete the Committee.

In addition to the foregoing, provisions are made for voluntary communications, by the following resolutions:

IV. *Resolved*, That a Committee of five members be appointed, to be called the Committee for Volunteer Communications, whose duty it shall be, in the interval between the present and the next succeeding sessions, to receive papers upon any subject from any persons who may choose to send them, to decide upon the merits of these papers, and to select for presentation to the Association, at its next session, such as they may deem worthy of being thus presented.

V. *Resolved*, That the Committee for Volunteer Communications, shall have the power to form such regulations as to the mode in which the papers are to be presented, and as to the observing of secrecy, or otherwise, as they may think proper.

VI. *Resolved*, That the selection of the members of the Committee be referred to the same Nominating Committee, whose duty it will be to appoint the Chairmen of the several Special Committees, as above directed; with this restriction, that the individuals composing it shall reside in the same neighborhood.

VII. *Resolved*, That a prize of fifty dollars be awarded to each of the Volunteer Communications reported on favorably by the Committee, and directed by the Association to be published: Provided, that the number to which the prize is thus awarded, do not exceed five; and provided, also, if the number approved and directed to be published exceed five, that in such case, the prize be awarded to the five which the Committee may determine to be most meritorious. All of which is respectfully submitted.

GEORGE B. WOOD, Chairman.

The following gentlemen were appointed on the Committee for Volunteer Communications, viz: Dr. George Hayward, J. B. S. Jackson, D. H. Storer, and Jacob Bigelow, of Boston, and Dr. Usher Parsons, of Providence, R. I.

We know not how this arrangement will strike the minds of members of the profession generally, but, in our estimation, the change is unfortunate. We may find reasons to entertain a different opinion, and shall certainly try to think that the alteration is for the best, if it is to be permanent. So great a change should not have been entered upon without careful consideration, which, we presume, was bestowed upon the subject by those who were instrumental in bringing it about. As we now think, it will fail to enhance the interest and usefulness of the Association. The effect of so sudden and radical an alteration cannot but be bad, unless a very decided improvement is to be effected, because it gives an idea of mutability, and conveys the impression that the working of the Association, thus far, has been unsatisfactory. The reason stated for the change in the communication by Dr. Parkman, in the Boston Medical Journal, is, that reports, such as the Association has heretofore been accustomed to receive, must, necessarily, for the most part, be a retrospect, or compendium gathered from the various medical journals. Now, this, in our humble opinion, is precisely what is to be desired, and what will most conduce to the value and number of original contributions during the year. The profession wish to know what is doing in the various portions of the country, in the several departments of medical science. It is to the medical journals, to the transactions of State and local societies, &c., that we are to look primarily for new developments in medicine, surgery, midwifery, &c. They are the most convenient and proper channels for proposed improvements to come before the medical public. An annual abstract of whatever is to be derived from these sources, worthy of attention, will, assuredly, present the fairest and fullest view of progress in the various departments mentioned, during each year. And this is wanted, for encouragement at home, and for the credit of our national character, both at home and abroad. The published transactions should be, as we think, the exponent of what has been done in the different sections of the United States during the year. In this way we shall be led to do ourselves justice, and shall have justice done to us by others. Moreover, by a reflected influence, members of the profession throughout the country, will be incited thereby to contribute more and more to the stock of medical knowledge. The periodical literature of the country will be encouraged, and this is an object which, in view of its great importance, should not be lost sight of.

These are some of the considerations which lead us to regard in an unfavorable light the new plan that has been adopted by the Association. This plan proposes to give out a series of stated topics, like themes to a class of college students; and, in the meantime, articles that may appear in the Journals, or elsewhere, of ever so much value, and calculated to do ever so much credit to the country, must remain unnoticed, unless the authors are willing to compete for a prize. We are perhaps entirely mistaken in these views, and, if so, we shall be very glad to be undeceived.

The subjects that have been designated appear to us obnoxious to criticism on the score of their being only indirectly of a practical character. They are, moreover, too numerous. Is it probable that *twenty-seven* dissertations, with an indefinite number of voluntary contributions, will be read during the session of the Association? But we will waive further comment on the matter at this time, with the design of resuming it hereafter.

The prize offered at the former annual meeting for the best "*Experimental* essay on a subject, connected either with Physiology, or Medical Chemistry,"

was awarded to Dr. John C. Dalton, junior, of Bostcn, Mass., the newly appointed professor of Physiology in the Medical School of this city. The Boston Medical and Surgical Journal thus comments on this fact: "As one of the gratifying circumstances of the present meeting, and in which our city may take pride, may be mentioned, that the prize offered a year since, was obtained by Dr. John C. Dalton, junior, of Boston, for a treatise on Ovology. This paper was spoken of to the writer of this notice, before the name of the successful author was known, as of a very remarkable character, and as being accompanied by drawings in a very high style of art. It will appear by a vote of the Association, among its transactions."

Richmond, Va., was selected as the place for the annual meeting in 1852.

